# MICRObiota on BILIOpancreatic malignant diseases [MICROBILIO]: A systematic review

**DOI:** 10.1016/j.clinsp.2022.100101

**Published:** 2022-09-17

**Authors:** Vitoria Carneiro de Mattos, Fernanda Sayuri do Nascimento, Milena Oliveira Suzuki, João Victor Taba, Leonardo Zumerkorn Pipek, Walter Augusto Fabio Moraes, Vitor Santos Cortez, Márcia Saldanha Kubrusly, Matheus Belloni Torsani, Leandro Iuamoto, Wu Tu Hsing, Luiz Augusto Carneiro-D'Albuquerque, Alberto Meyer, Wellington Andraus

**Affiliations:** a Faculdade de Medicina, Universidade de São Paulo (FMUSP), São Paulo, SP, Brazil; b Department of Gastroenterology (LIM-37), Faculdade de Medicina, Universidade de São Paulo (FMUSP), São Paulo, SP, Brazil; c Center of Acupuncture, Department of Orthopaedics and Traumatology, Faculdade de Medicina, Universidade de São Paulo (FMUSP), São Paulo, SP, Brazil; d Department of Gastroenterology, Hospital das Clínicas, Faculdade de Medicina, Universidade de São Paulo (HCFMUSP), São Paulo, SP, Brazil

**Keywords:** Pancreatic Neoplasms, Microbiota, Early detection of cancer, Dysbiosis, Tumor microenvironment, Biliary tract neoplasms, Gastrointestinal microbiome

## Abstract

**Introduction:**

The increase in the incidence of pancreatic and biliary cancers has attracted the search for methods of early detection of diseases and biomarkers. The authors propose to analyze new findings on the association between microbiota and Pancreatic Ductal Adenocarcinoma (PDAC) or Cholangiocarcinoma (CCA).

**Methods:**

This systematic review was carried out according to the items of Preferred Reports for Systematic Reviews and Protocol Meta-Analysis (PRISMA-P). This study was registered by the Prospective Register of Systematic Reviews (PROSPERO), identification code CRD42020192748 before the review was carried out. Articles were selected from the PUBMED, EMBASE, and Cochrane databases.

**Results:**

Most studies (86.67%) used 16s rRNA as a sequencing method. The main comorbidities found were diabetes mellitus, systemic arterial hypertension, and dyslipidemia. Many studies were limited by the small number of participants, but the biases were mostly low. There was very little concordance about the composition of the microbiome of different sites, for both case and control groups when compared to other studies' results. Bile sample analysis was the one with a greater agreement between studies, as three out of four studies found *Escherichia* in cases of CCA.

**Conclusion:**

There was great disagreement in the characterization of both the microbiota of cases and control groups. Studies are still scarce, making it difficult to adequately assess the data in this regard. It was not possible to specify any marker or to associate any genus of microbiota bacteria with PDAC or CCA.

## Highlights

• Microbial agents and their metabolites are being tested to develop treatments that can reduce the tumor and are potentially preventable.
• Microbiota is observed in all clinical and pathological stages of carcinogenesis, from its development, diagnosis, and treatment, including prognosis and survival.
• There is a lack of studies on biliary microbiota and its relationship with hepatobiliopancreatic diseases.

## Introduction

The incidence and prevalence of cancer have increased over time.^1^ Pancreatic cancer, mainly Pancreatic Ductal Adenocarcinoma (PDAC), is the fourth type of cancer with the highest overall mortality in the United States^2^ and, in 2018, was responsible for about 4.6% of cancer deaths worldwide,^3^ with 4.8% in Brazil.^4^ Bile duct cancer has a low incidence in the Western world (between 0.35 and 2 per 100,000 per year). Due to the initial silent progression and the difficulty of early detection, both diseases have a poor prognosis, with five years survival of less than 20%.^5^^,^^6^ Screening is restricted to individuals at high risk of developing the diseases,^7^ which are often discovered in advanced, metastatic stages, as there are no large-scale or non-invasive screening tests available.

The etiology of these cancers is still not well defined. Among the most mentioned causes, there are chronic inflammation, genomic factors, biliary cysts, viral infections and, recently, alterations in the microbiota, called dysbiosis.^8^

Despite the difficulty in determining what constitutes a microbiota in eubiosis or dysbiosis, some associations can be established. Del Castillo et al.,^9^ found a reduction in the presence of *Lactobacillus* and an increase in *Porphyromonas* in cases of PDAC compared to the control group. Wei M-Y et al. found an association of *Porphyromonas gingivalis*, which causes chronic periodontitis, with the development of PDAC, through the expression of peptidyl-arginine-deiminase, which promotes mutations in K-ras and p53. The relationship between the presence of *Helicobacter pylori*, a bacterium associated with malignant transformation in the stomach, and the development of PDAC is also being studied. However, its participation as a risk factor for the disease is not yet proven, in addition to being very controversial.^10^, ^11^, ^12^, ^13^

Regarding CCA, despite the small number of studies on the subject, some demonstrate that the presence of *H. pylori, H. bilis and H. hepaticus* in the intestine leads to an increase in the Nuclear Factor Kappa B (NFKB) and nuclear signaling pathway production of Vascular Endothelial Growth Factor (VEGF). Thus, there would be an increased risk for the development of neoplasms, with angiogenesis promotion in the tumor site.^14^

When considering the relationship between cancer and changes in the microbiota, there is a possibility of tracking the disease. Farrel et al.,^10^ after finding a reduction in bacteria from the oral microbiota, *Neisseria elongata* and *Streptococcus mitis*, in patients with PDAC, suggested that studying the oral microbiota could be used as screening for pancreatic neoplasms. Thus, there are a variety of sites to be potentially explored for a better understanding of this disease, not only the pancreas itself.

The mechanism of colonization of the bile ducts and pancreas is not yet defined and is still a topic under discussion.^15^^,^^16^ Given the high morbidity and mortality of PDAC and CCA and the difficulties of early detection, it is essential to develop methods for screening the disease and discovering biomarkers.

## Objective

The aim of this systematic review is to evaluate new findings and reports on the composition of the gastrointestinal tract microbiota in cases of pancreatic and biliary cancer.

## Methods

This systematic review was carried out according to the items of Preferred Reports for Systematic Reviews and Protocol Meta-Analysis (PRISMA-P).^17^ This study was registered by the Prospective Register of Systematic Reviews (PROSPERO, identification code CRD42020192748) before the review was carried out.

The preparation of the research question was based on the PICO strategy,^18^ considering diseases of the pancreas and biliary tree (Patient or Problem); microbiota impact (Interest); healthy people or people with benign diseases (Control or Comparison); all outcomes available in the literature were c onsidered in the analysis (Outcome).

### Eligibility criteria

#### Types of studies

Articles were selected from their titles and abstracts according to their data relevance and regardless of their publication status. Articles with full text inaccessible to authors were not considered.

The following study designs were considered: randomized controlled clinical trials, non-randomized controlled clinical trials, prospective and retrospective cohorts, case-control and cross-sectional. Reports and case series, reviews, letters to the editors, research protocols and congress proceedings were not considered.

#### Types of participants

Study participants were adults with pancreatic or bile duct cancer and control subjects who underwent gastrointestinal microbiota evaluation.

#### Methods of sample collection

The sample collection strategies evaluated in the study are stool analysis, ERCP, oral swab, surgery for biliopancreatic disease and upper digestive endoscopy.

#### Types of variables/parameters analyzed

Data relating to the authors, date, and location (country) of publication, type of study, analysis methods, analyzed site, associated factors and microbiota alteration were collected and arranged in tables.

### Exclusion criteria

Studies were excluded if: samples were collected from children, adolescents, patients with autoimmune disease, cadavers, or rodents; the article is incomplete or unpublished; tumors that are not primarily of the pancreas or biliary tree; are in other languages except English and Portuguese.

### Literature review

The survey was conducted on August 11, 2021, without language or date restrictions, in the following databases: Medline (via PubMed) ‒ www.pubmed.com; EMBASE ‒ www.embase.com and Cochrane ‒ www.cochranelibrary.com.

Using the PubMed search tool, the authors selected MeSH terms from the most relevant publications to perform a new search, in order to obtain articles that could be included in this systematic review.

In addition, a manual search of theses, meetings, references, study records, and contact with experts in the field was carried out.

#### Search strategy

The keywords were used equally in all databases, respecting their heterogeneities (for example, Emtree terms and MeSH terms were mapped in Embase and Medline, respectively).

The keywords were: “pancreatic neoplasms”, “microbiota”, “early detection of cancer”; “dysbiosis”, “tumor microenvironment” and “biliary tract neoplasms”.

The search strategy was: ((Microbiota) or (Dysbiosis)) and ((Pancreatic Neoplasms) or (Biliary Tract Neoplasms) or (Early Detection of Cancer) or (Tumor Microenvironment)).

#### Data extraction

Data from each study were independently extracted by four authors (V.C.M., E.M.C.D., M.O.S and F.S.N.). Disagreements were resolved by consensus. If no consensus was reached, a fifth author (A.M.) would be consulted. Data extraction was carried out using the Rayyan tool ‒ https://rayyan.qcri.org/.^19^

All studies were analyzed according to their titles and abstracts, according to inclusion and exclusion criteria. If the eligibility criteria were met, the full text would be extracted. All evaluated full-text studies were described in the “Results” section.

Missing data were clarified by contacting the authors directly.

#### Data validation

The four authors performed data validation through the discussion of selected works. If no consensus was reached, a fifth author would be consulted.

The risks of bias for the studies were assessed using the Study Quality Assessment Tools | National Heart, Lung, and Blood Institute (NHLBI).^20^ Intervention-type studies were analyzed using the guidelines of the Cochrane Back Review Group (CBRG).^21^

All selected studies were considered.

#### Authors' responsibilities/contributions

V.C.M., M.O.S. and F.S.N.: Conception, methodology, formal analysis, investigation, writing, drafted the work; J.V.T. and L.Z.P.: Validation, review; AM: Conception, methodology, investigation, supervision, project administration.

All authors have approved the submitted version and have agreed to both be personally accountable for the author's own contributions and to ensure that questions related to the accuracy or integrity of any part of the work, even ones in which the author was not personally involved, are appropriately investigated, resolved, and the resolution documented in the literature.

## Results

### Search flow

**Fig. 1:**
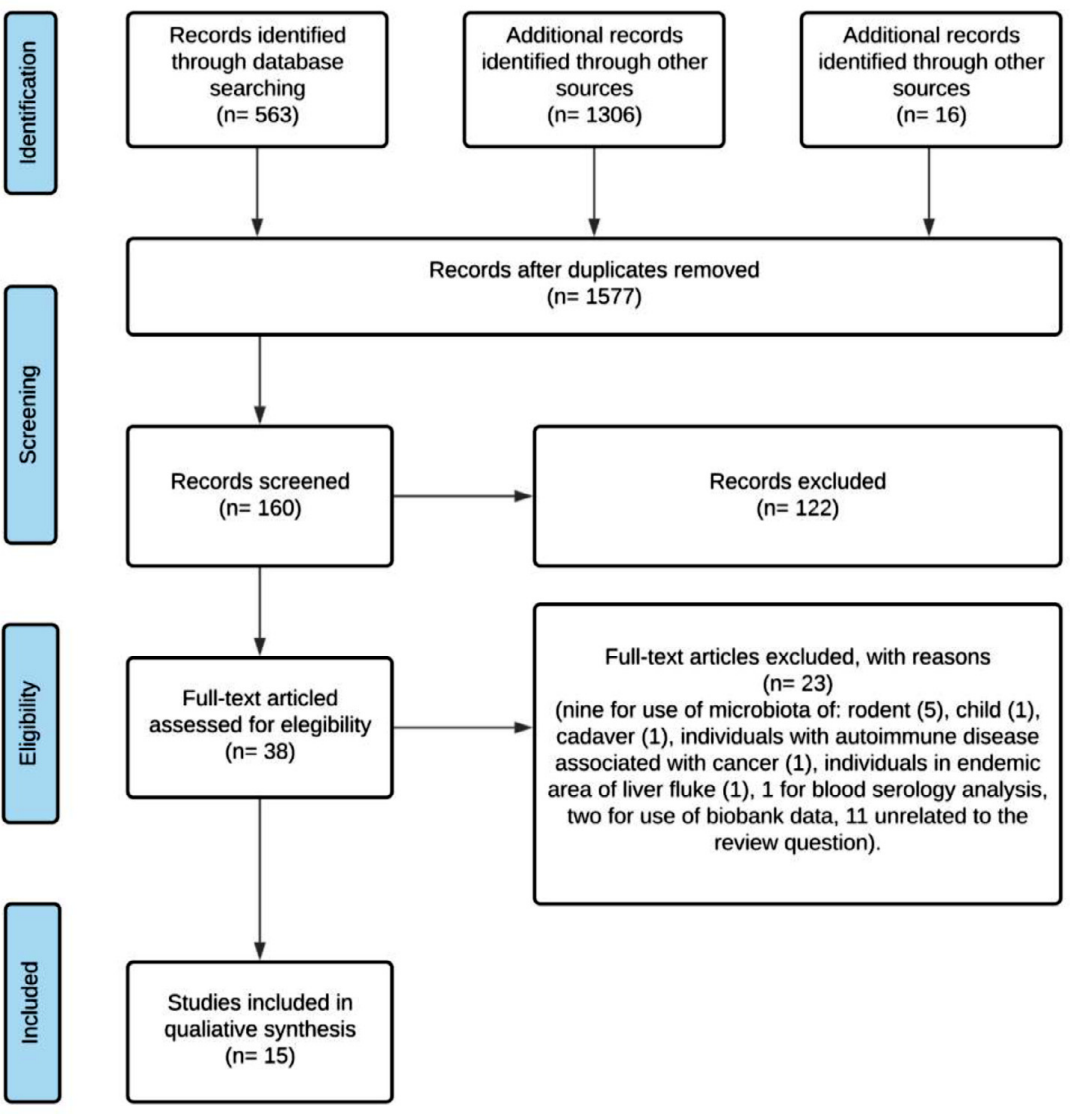
Search flow.

1577 results were found for the keywords used. A total of 308 duplicates were removed, leaving 160 potentially eligible studies after abstract analysis. Of those selected, 122 were not included for not meeting the inclusion criteria, 5 studies were excluded for using rodents, 1 study for inclusion of children, 1 for using a cadaver, 1 for treating an autoimmune disease associated with cancer, 1 for study in a liver fluke endemic area, 1 for blood serology analysis, 2 for use of biobank material and 11 not related to the review question. In the end, 15 studies were selected for qualitative analysis (Fig. 1).

### Limitations and methodologies of studies

Among the limitations reported in the studies, the small size of the population sample, the single collection of the material to be analyzed, and collection after cancer diagnosis were the most frequent (Table 1).

**Table 1:** Reported studies limitations.

Author, publication date and country	Reported studies limitations
Chen B (2019), China	Not reported
Vogtmann E (2020), USA	1. Saliva sample collected after cancer diagnosis
	2. Unhealthy controls (benign diseases)
	3. Low power study for rate-specific analysis
	4. Information on oro-dental health was not obtained
Half E (2019), Israel	1. Small sample
Serra N (2018), Canada	Not reported
Olson SH (2017), USA	1. Small sample
	2. One-time collection of oral samples
Di Carlo P (2019), Italy	1. Study at a single center
Mei Q-X (2018), China	1. Small sample
	2. Analysis of bacteria only
Erick R (2019), USA	Not reported
Torres PJ (2015), USA	1. Low power study for rate-specific analysis of *Streptococcus*
	2. Presence of tumor beyond the primary site (pancreas)
Ren Z (2017), China	1. Stool sample collected after cancer diagnosis
Fan X (2018), USA	1. One-time collection of oral samples
	2. Information on oro-dental health was not obtained
	3. Unrepresentative groups of the general population, low generalization power
Kohi S (2021), USA	1. Study at a single center
	2. Presence of confounding factors in cases and controls
	3. Duodenal fluid sample collected after cancer diagnosis
Saab M (2021), France	1 Unhealthy control (benign diseases) and no literature for comparison
	2. No comparison with tumor microbiota
	3. Possibility of contamination of samples with duodenal bacteria
Sun H (2020), China	Not reported
Wei AL (2020), China	1. Non-inclusion of other pancreatic diseases
	2. Only 16S rRNA sequencing for analysis
	3. Information on oro-dental health was not obtained

Fan, X et al.^22^ used saliva samples collected prior to suspicion and diagnosis of PDAC. Thus, results were obtained without the potential influence of the disease on the microbiome composition. However, post-diagnosis samples were not collected to identify whether there are changes in the microbiota caused by this condition.

### Quality of evidence

**Fig. 2:**
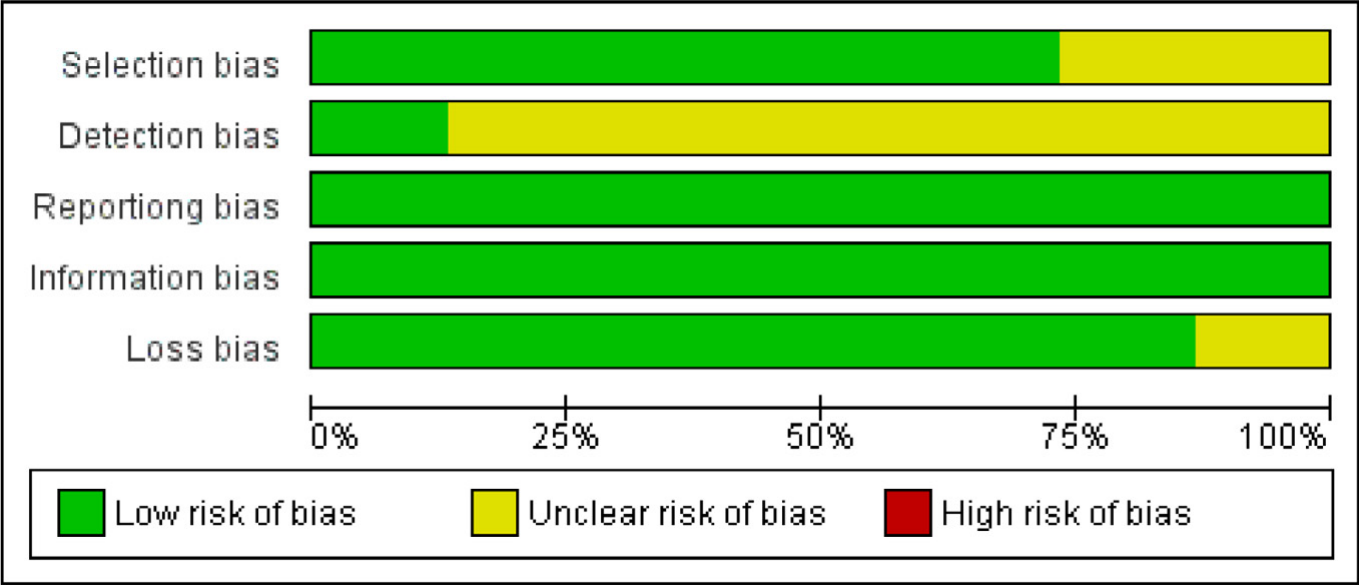
Graph of risk analysis of general bias in articles.

**Fig. 3:**
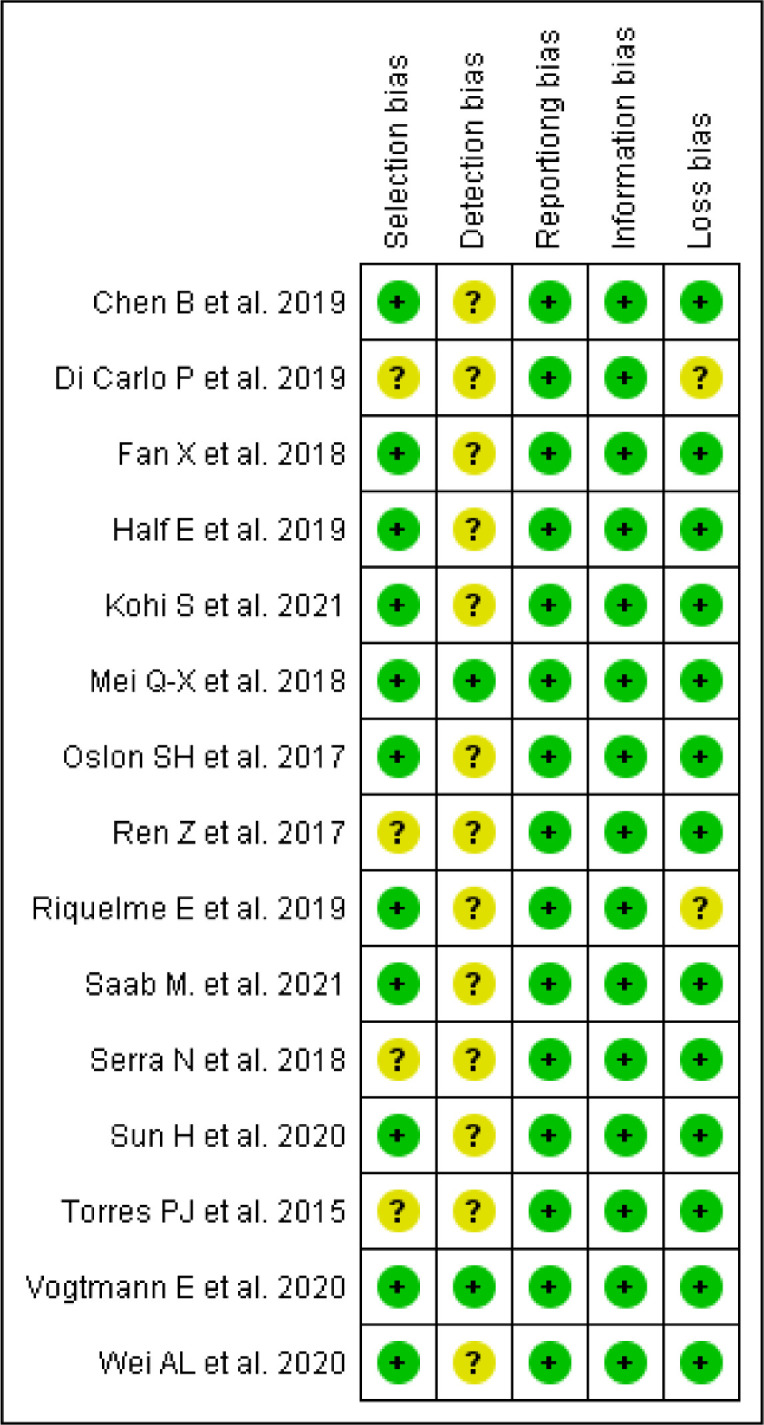
Summary of risk analysis of general articles bias.

The articles selected for this review are studies that collect material (saliva, feces, bile, tissue, and duodenal and bile duct fluid) from patients and controls, and analyze the microbiota present in each sample. After analyzing the studies, the selection, detection, reporting, information, and loss biases were observed to define the quality of the evidence found, with the classification displayed in Figs. 2 and 3.

The studies included in this review show mostly low selection bias, with 11 being low and 4 uncertain. Among the uncertain case-control studies, Torres, P.J. et al.,^23^ determines the ethnicity of its participants but do not restrict the location of the population in question. Ren, Z. et al.^24^ describes the country of origin analyzed, but does not describe ethnic and socioeconomic characteristics.

The cross-sectional study by Chen, B. et al.^25^ and the cohort of Riquelme E., et al.^26^ had low selection bias. The first one selected and recruited patients from the same population, that underwent the ERCP procedure between 2016 and 2017 at the Shanghai General Hospital, and both specified and uniformly applied inclusion and exclusion criteria in the selection.

The cross-sectional study by Serra, N. et al.^27^ and the cohort of Di Carlo, P. et al.^28^ had an uncertain selection bias, as it did not report the exclusion criteria, and it was not possible to determine whether these were applied uniformly to all participants.

Furthermore, the studies present an uncertain detection bias, as they do not report whether or not there was a blinding in the performance of the analyses. This cannot be applied to Vogtmann, E. et al.^29^ and Q.-X. Mei et al.,^30^ showed low detection bias. Vogtmann, E. et al. performed a blinded analysis of each sample, whether case or control, specifying this in the methodology. Q.-X. Mei et al., however, did not clarify regarding the blinding in the analysis of the microbiome of each study participant, only the blinding of pathologists in the histological analysis of duodenal tissue samples.

As the composition of the microbiome is a non-self-reported characteristic, without the possibility of being influenced by factors such as patient memory, omission, or addition of data, all articles analyzed in this review had low reporting bias.

The low information bias, shared by all of the articles considered, is due to the clear criteria used to separate the groups. The division was made based on the histopathological diagnosis of the presence or absence of cancer, reducing the wrong allocation.

The low loss bias remains on the fact that most studies performed a single analysis of the microbiome without the need for follow-up, both with patients and controls. However, two cohorts showed unclear loss bias (Di Carlo, P. et al.^28^ and Riquelme E. et al.^26^), due to the fact that both analyze the survival of selected patients. Thus, for this review, only the data collected at admission were used.

### Characteristics of the studies

The demographic characteristics collected were displayed in Table 2; the main changes, results and conclusions are provided in Table 3, Table 4, Table 5.

**Table 2:** Demographic characteristics of studies.

Author, publication date and country	Number of patients	Mean age ‒ years (SD) or Range of age (n)	Sex (%)	Associated factors (n or %)
Chen B (2019), China	CCA: 8	CCA: 72.13 (range: 60‒95)	CCA: 3 M (37.5); 5 F (62.5)	DM: CBD stones: 10, RC: 4
	CBD stones: 44	CBD stones: 66.98 (range: 44‒90)	CBD stones: 18 M (40.9); 26 F (59.1)	Dyslipidemia: CCA: 1, CBD stones: 15, RC: 4
	RC: 16	RC: 73.94 (range: 49‒92)	RC: 9 M (56.2); 7 F (43.8)	Hypertension: CCA: 5, CBD stones: 17, RC: 6
	Total: 68			Elevated ALT or AST: CCA: 7, CBD stones: 25, RC: 7
				Elevated Tbil and/or Dbil: CCA: 6, CBD stones: 19, RC: 7
				Elevated Scr: CCA: 2, CBD stones: 6, RC: 2
				Elevated WBC or NE: CBD stones: 7
				Cholecystolithiasis: CCA: 1, CBD stones: 23, RC: 7
Vogtmann E (2020), USA	PDAC: 273	PDAC: < 50 (26), 50‒59 (61), 60‒69 (87), 70‒79 (77), ≥ 80 (22)	PDAC: 165 M (60.4), 108 F (39.6)	‒
	Control group: 285		Control group: 131 M (46); 154 F (54)	
		Control group: < 50 (20), 50‒59 (78), 60‒69 (94), 70‒79 (59), ≥ 80 (34)		
	Total: 558			
Half E (2019), Israel	Case group: PDAC: 30, PCL: 6	PDAC: 69.9 (6.2)	PDAC: 16 M (53.5), 14 F (46.7)	DM: PDAC: 53%, PCL: 20%, NAFLD: 13%
	Control group: NAFLD: 16	PCL: 66 (15.3)	PCL: 5 M (83.3), 1 F (16.7)	Hypertension: PDAC: 43%, PCL: 25%, NAFLD: 50%
	Healthy control: 13	NAFLD: 51 (10.8)	NAFLD: 12 M (75), 4 F (25)	
	Total: 65	Healthy control: 59 (8.7)	Healthy control: 6 M (46.6), 7 F (53.4)	Dyslipidemia: PDAC: 40%, PCL: 29%, NAFLD: 88%, Healthy control: 23%
				Bile-duct obstruction: PDAC: 36%
				Gall-bladder abnormalities: PDAC: 46%, NAFLD: 6%, Healthy control: 23%
Serra N (2018), Canada	CCA: 20, GBC: 2, PDAC: 31, Total: 53	73.4 (10.5)	0 M (0), 53 F (100)	Intra-abdominal infection: 53
Olson SH (2017), USA	PDAC: 40^a^	PDAC: < 60 (10), 60‒69 (12), ≥ 70 (12)	PDAC: 18 M (53), 16 F (47)	DM: PDAC: 9, IPMN: 4, Healthy control group: 7
	IPMN: 39			
	Healthy control group: 58	IPMN: < 60 (8), 60‒69 (8), ≥ 70 (23)	IPMN: 22 M (40), 17 F (60)	Álcool: PDAC: 25, IPMN: 36, Healthy control group: 53
	Total: 137	Healthy control group: < 60 (20), 60‒69 (7), ≥ 70 (11)	Healthy control group: 23 M (56), 35 F (44)	Gum disease ever: PDAC: 14, IPMN: 15, Healthy control group: 19
Di Carlo P (2019), Italy	PDAC: 72	PDAC: 75.6 (10.4)	PDAC: 41 M (56.9), 31 F (43.1)	‒
	CCA: 39			
	Total: 111	CCA: 71.5 (8.8)	CCA: 19 M (48.7), 20 F (51.3)	
Mei Q-X (2018), China	PDAC: 14	PDAC: 56.8 (5.1)	PDAC: 9 M (64.3), 5 F (35.7)	‒
	Control group: 14			
		Control group: 55.4 (6.2)	Control group: 9 M (64.3), 5 F (35.7)	
	Total: 28			
Riquelme E (2019), USA	PDAC Discovery cohort (DC): LTS: 21, STS: 22	DC: LTS: 62.71 (range: 44‒73), STS: 62.05 (range: 46‒74)	DC: LTS: 10 M (47.62), 11 F (52.38); STS: 13 M (59.1); 9 F (41.9)	‒
	Validation cohort (VC): LTS: 15, STS: 10			
Torres PJ (2015), USA	PDAC: 8	PDAC: 71.1	PDAC: 6 M (75), 2 F (25)	Other diseases group: Non-pancreatic cancer, Pancreatic diseases (not cancer)
	Others diseases: 78	Others diseases: no descript		
			Others diseases: 38 M (48.72), 40 F (51.28)	
	Healthy control group: 22,	Healthy control group: no descript		
			Healthy control group: 12 M (54.55), 10 F (45.45)	
	Total: 108			
Ren Z (2017), China	PDAC: 85	PDAC: 56 (range: 33‒78)	PDAC: 47 M (55.3), 38 F (44.7)	‒
	Healthy control group: 57	Healthy control group: 52 (range: 43‒67)	Healthy control group: 36 M (63.2), 21 F (36.8)	
	Total: 142			
Fan X (2018), USA	Group 1: PDAC: 170, Control group: 170	Group 1: PDAC: 73.7 (5.7), Control group: 73.7 (5.7)	Group 1: PDAC: 90 M (52.9), 80 F (47.1), Control group: 90 M (52.9), 80 F (47.1)	‒
	Group 2: PDAC: 191, Control group: 201	Group 2: PDAC: 63.8 (5.2), Control group: 633.8 (5.4)	Group 2: PDAC: 116 M (60.7), 75 F (39.3), Control group: 122 M (60.7), 79 F (39.3)	
	Total: 732			
Kohi S (2021), USA	Control: 134	Control: 63.6 (41.6–79.5)	Control: 30 M (47.6%), 33 F (52.4%)	‒
	PDAC: 74			
	Cyst: 98	PDAC: 42.2–85.5	PDAC: M 64%, F 36%	
	Total: 308	Cyst: 65.8 (42.9–87.8)	Cyst: 36 M (50%), 36 F (50%)	
Saab M (2021), France	CCA: 28	CCA: 64 (12)	CCA: 19 M (68), 9 F (32)	DM: CCA: 6, Biliary duct lithiasis: 9
	Biliary duct lithiasis: 47	Biliary duct lithiasis: 57 (17)	Biliary duct lithiasis: 23 M (49), 24 F (51)	Pancreatitis: CCA: 0, Biliary duct lithiasis: 1
	Total: 75			Inflammatory bowel disease: CCA: 2, Biliary duct ithiasis: 0
				Primary sclerosing cholangitis: CCA: 1, Biliary duct lithiasis: 0
Sun H (2020), China	PDAC: 10	PDAC: 57.4 (7.8)	PDAC: 6 M (60), 4 F (40)	Alcool: PDAC: 2, BPD: 3, Healthy control group: 1
	BPD: 17	BPD: 42.8 (16)		
	Healthy control group: 10	Healthy control group: 31.1 (2.7)	BPD: 10 M (58.82), 7 F (41.17)	Smoking: PDAC: 2, BPD: 3, Healthy control group: 2
	Total: 37		Healthy control group: 6 M (60), 4 F (40)	BPD: Pancreatitis, chronic pancreatitis, benign pancreatic tumors
Wei AL (2020), China	PDAC: 41	PDAC: 61.17 (1.79)	PDAC: 24 M (59), 17 F (41)	DM: PDAC: 5, Healthy control group: 11
	Healthy control group: 69	Healthy control group: 64.64 (1.04)	Healthy control group: 50 M (72), 19 F (28)	Hypertension: PDAC: 4, Healthy control group: 20
	Total: 110			Alcool: PDAC: 16, Healthy control group: 30
				Smoking: PDAC: 17, Healthy control group: 37

M, Male; F, Female; CBD stones, Common bile duct stones; RC, Recurrent Choledocholithiasis; PC, Pancreatic Cancer; PCL, Precancerous Lesions; NAFLD, Non-alcoholic Fat Liver Disease; CCA, Cholangiocarcinoma; GBC, Gallbladder Carcinoma; PDAC, Pancreatic Ductal Adenocarcinoma. IPMN, Intraductal Papillary Mucinous Neoplasms; DM, Diabetes Mellitus; AST, Aspartate Aminotransferase; ALT, Alanine Aminotransferase; Tbil, Total Bilirubin; Dbil, Direct Bilirubin; Scr, Serum Creatinine; WBC, White Blood Cell; NE, Neutrophilic Granulocyte.

a 6 of the patients with PDAC did not complete the questionnaire (total number of responses: 131), but samples of the microbiome were collected and included in the analysis.

**Table 3:** Experimental studies main results.

Author, publication date and country	Study type	Analysis Method	Type of disease	Type of sample	Microbiota (caso/controle)	
Vogtmann E (2020), USA	Case-control	16s rRNA	Case: PDAC	Saliva	Phylum: *Bacteroidetes, Firmicutes, Proteobacteria*	Phylum*: Proteobacteria*
			Control: Submucosal lesions in the esophagus or stomach, Cholelithiasis or choledocholithiasis without cholangitis.			Family*: Pasteurellaceae*
					Family: *Bacteroidaceae, Staphylococcaceae, Enterobacteriaceae, Lachnospiraceae*	Genus*: Haemophilus*
					Genus: *Lachnospiraceae* G7	
Half E (2019), Israel	Case-control	16s rRNA	PDAC	Stool	PDAC (compared with healthy individuals from the Israeli cohort): Phylum: *Firmicutes, Verrucomicrobia, Bacterioidetes*, Family: *Veillonellaceae, Akkermansiaceae, Odoribacteraceae*, Genus: *Megasphaera, Akkermansia, Odoribacter*	Healthy (compared to individuals with PDAC in the Israeli cohort): Phylum: *Firmicutes, Tenericutes*, Family: *Clostridiacea, Lachnospiraceae*, Erysipelotrichaeceae, *Ruminococcaceae*, Genus: *Clostridium, Anaerostipes, Faecalibacterium, Subdoligranulum*
					PDAC (compared to individuals with PDAC in the Chinese cohort): Phylum: *Firmicutes*, Family: *Veillonellaceae*	Healthy (compared to individuals with PDAC in the Chinese cohort): Phylum: *Firmicutes*, Family: *Erysipelotrichaeceae, Clostridiaceae*, Genus: *Anaerostipes*
Olson SH (2017), USA	Case-control	16s rRNA	Case: PDAC	Saliva	PDAC (compared to healthy control or IPMN): Phylum: *Firmicutes*, Family: *Streptococcaceae*, Genus: *Streptococcus*	Healthy control (compared with PDAC): Phylum: *Proteobacteria*, Family: *Pasteurellaceae, Neisseriaceae*, Genus: *Haemophilus, Neisseria*
			Control: IPMN			
Mei Q-X (2018), China	Case-control	16s rRNA	PDAC	Duodenum	PDAC (compared to healthy control): Phylum: *Proteobacteria, Firmicutes, Deinococcus-Thermus*; Family: *Moraxellaceae, Comamonadaceae, Yersiniaceae, Comamonadaceae, Sphingomonadaceae, Bacillaceae, Deinococcaceae, Oxalobacteraceae*; Genus: *Acinetobacter, Aquabacterium, Rahnella, Delftia, Sphingobium, Massilia, Oceanobacillus, Deinococcus*	Control (compared to PDAC): Phylum: *Proteobacteria, bacterioidetes, firmicutes*; Family: *Enterobacteriaceae, Pseudomonadaceae, Incertae sedis, Phophyromonadaceae, Paenibacillaceae*; Genus: *Escherichia, Shigella, Pseudomonas, Enhydrobacter, Porphyromonas, Paenibacillus*
					Most Abundant PDAC: Phylum: *Firmicutes, Proteobacteria, Bacteroidetes*; Family: *Bacillaceae, Pseudomonadaceae, Streptococcaceae*; Genus: Bacillus, *Pseudomonas, Lactococcus*	More abundant healthy: Phylum: *Firmicutes, Proteobacteria, Bacteroidetes*; Family: *Bacillaceae, Pseudomonadaceae, Streptococcaceae*; Genus: *Bacillus, Pseudomonas, Lactococcus*
Torres PJ (2015), USA	Case-control	16s rRNA	PDAC	Saliva	Increase in PDAC (compared to other diseases and healthy controls): Phylum: *Fusobacteria*, Family: *Leptotrichiaceae*, Genus: *Leptotrichia*	No descript
					Decrease in PDAC (compared to other diseases and healthy controls): Phylum: *Bacteroidetes, Proteobacteria*, Family: *Porphyromonadaceae, Neisseriaceae*; Genus: *Porphyromonas, Neisseria*	
Ren Z (2017), China	Case-control	16s rRNA	PDAC	Stool	PDAC (compared with healthy control group): Phylum: *Bacteroidetes*; Family: *Prevotellaceae, Veillonellaceae, Enterobacteriaceae*; Genus: *Prevotella, Hallella, Veillonella, Selenomonas, Klebsiella, Enterobacter, Cronobacter*	Healthy control group (compared with PDAC): Phylum: *Firmicutes, Proteobacteria, Actinobacteria*; Family: *Ruminococcaceae, Lachnospiraceae, Clostridiaceae, Bifidobacteriaceae*; Genus: *Gemmiger, Flavonifractor, Coprococcus, Blautia, Anaerostipes, Clostridium IV, Butyricicoccus, Dorea, Bifidobacterium*
					Most Abundant PDAC: Phylum: *Bacteroidetes, Firmicutes* e *Proteobacteria*	More abundant healthy control group: Phylum: *Bacteroidetes, Firmicutes* e *Proteobacteria*
Fan X (2018), USA	Case-control	16s rRNA	PDAC	Saliva	Associated with high risk of pancreatic cancer: Phylum: *Bacterioidetes1, Proteobacteria2*; Family: *Porphyromonadaceae1, Pasteurellaceae2*; Genus: *Porphyromonas1, Aggregatibacter2*; aEspécie: *Porphyromonas gingivalis1, Aggregatibacter actinomycetemcomitans2*	Associated with low risk of pancreatic cancer: Phylum: *Fusobacteria*; Family: *Leptotrichiaceae*; Genus: *Leptotrichia*
Kohi S (2021), USA	Case-control	16s rRNA	PDAC	Duodenal fluid	Most Abundant PDAC: Phylum*: Proteobacteria*	*Phylum: Firmicutes, Bacteroidetes, Proteobacteria: Class: Bacilli, Bacteroidia, Negativicutes, Gammaproteobacteria; Order: Lactobacillales, Bacteroidales, Selenomonadales; Familia: Streptococcaceae, Veillonellaceae, Prevotellaceae*; Genus*: Streptococcous, Veillonella, Prevotella 7*
			Pancreatic cyst			
					PDAC (compared with healthy control group): Phylum*: Actinobacteria, Fusobacteria, Firmicutes*; Family*: Bifidobacteriaceae, Lactobacillaceae, Enterococcaceae*; Genus*: Bifidobacterium, Fusobacterium, Enterococcus*	
					PDAC (compared with pancreatic cyst): Phylum*: Proteobacteria, Bifidobacterium, Enterococcus*; Genus*: Escherichia-Shigella, Clostridium sensu strictu, Enterococcus, Bifidobacterium*	*Cyst: Family: Porphyromonadaceae, Corynebacteriaceae, Leptotrichiaceae; Genus: Streptococcus, Veillonella, Prevotella*
					STS: Class: *Fusobacteria, Actinobacteria, Betaproteobacteria*; Genus: *Fusobacterium, Rothia, Neisseria*	There was no difference between cyst and control
Saab M (2021), France	Case-control	16s rRNA	CCA	Bile	CCA (compared with biliary lithiasis): Phylum: *Bacteroidetes*; Family: *Bacteroidaceae*; Genuss: *Streptococcus, Bacteroides* e *Pyramidobacter*	Biliary lithiasis (compared with CCA): Phylum: *Firmicutes*; Family: *Clostridiaceae, Enterobacteriaceae, Fusobacteriaceae, Enterococcaceae*; Genus: *Clostriduim, Klebsiella, Fusobacterium e Enterococcus*
			Biliary lithiasis			
Sun H (2020), China	Case-control	16s rRNA	PDAC	Saliva	PDAC e BPD (compared with Healthy control group): Phylum: *Spirochaetes*; Family: no important variation	Healthy control group (compared with PDAC e BPD): Phylum: *Proteobacteria*; Family: *Neisseriaceae*
			BPD (benign pancreatic disease)			
					Most Abundant in PDAC cases: Phylum: *Bacteroidetes*; Family: *Prevotellaceae*, Genus: *Neisseria, Veillonella*	Most Abundant in Healthy control group: Phylum: *Proteobacteria*; Family: *Neisseriaceae*; Genus: *Neisseria*
Wei AL (2020), China	Case-control	16s rRNA	PDAC	Saliva	Increased in PDAC cases (compared with Healthy control group): Phylum: *Firmicutes, Fusobacteria, Actinobacteria, Proteobacteria*; Family: *Streptococcaceae, Lactobacillaceae, Leptotrichiaceae, Actinomycetaceae, Micrococcaceae, Enterobacteriaceae*; Genus: *Streptococcus, Lactobacillus, Leptotrichina, Actinomyces, Rothia, Escherichia*	Reduced in PDAC cases (compared with Healthy control group): Phylum: *Proteobacteria, Firmicutes, Bacteroidetes*; Family: *Neisseriaceae, Veillonellaceae, Porphyromonadaceae, Flavobacteriaceae, Prevotellaceae*; Genus: *Neisseria, Selenomonas, Porphyromnas, Tannerella, Capnocytophaga, Alloprevotella*
					Associated with high risk of pancreatic cancer: Phylum: *Firmicutes, Fusobacteria*; Family: *Streptococcaceae, Leptotrichiaceae*; Genus: *Streptococcus, Leptotrichia*	Associated with low risk of pancreatic cancer: Phylum: *Proteobacteria, Bacteroidetes*; Family: *Neisseriaceae, Prevotellaceae*; Genus: *Neisseria, Veillonella*

PDAC, Pancreatic Ductal Adenocarcinoma; IPMN, Intraductal Papillary Mucinous Neoplasms. STS, Short-Term Survival.

a The study emphasized the importance of the presence or absence or abundance of these species in the result.

**Table 4:** Observational studies main results.

Author, publication date and country	Type of study	Analysis method	Type of disease found	Type of sample	Microbiota found (case/control)	
Chen B (2019), China	Cross-sectional	16s rRNA	Case: CCA	Bile	*CCA: Phylum: Gemmatimonadetes, Nitrospirae, Chloroflexi, Latescibacteria, and Planctomycetes, Proteobacteria1, Firmicutes2, Actinobacteria3; Family: Enterobacteriaceae1, Staphylococcaceae2.1, Ruminococcaceae2.2, Microbacteriaceae3.1, Corynebacteriaceae3.2; Genus: Escherichia/Shigella1, Klebsiella1, Staphylococcus2.1, Unclassified_Enterobacteriaceae, and Faecalibacterium2.2, Okibacterium3.1, and Corynebacterium3.2*	CBD stones: Phylum: *Proteobacteria1, Firmicutes2*; Family: *Enterobacteriaceae1.1, Halomonadaceae1.2, Streptococcaceae2*; Genus: *Escherichia/Shigella1.1, Klebsiella1.1, Enterococcus1.1, Halomonas1.2, Streptococcus2*
			Control: CBD stones			
Serra N (2018), Canada	Cross-sectional	BD Phoenix system	CCA	Bile	PDAC: Phylum: *Proteobacteria*; Family: *Enterobacteriaceae*; Genus: *Klebsiella*	
			GBC			
			PDAC			
					*CCA:* Phylum*: Proteobacteria*; Family*: Pseudomonadaceae* e *Enterobacteriaceae*; Genus*: Pseudomonas e Escherichia*	
					GBC: Phylum: *Proteobacteria*; Family: *Pseudomonadaceae*; Genus: *Pseudomonas*	
Di Carlo P (2019), Italy	Cohort	BD Phoenix system or Vitek-2 System	CCA	Bile	PDAC: Phylum: Proteobacteria1; Family: Enterobacteriaceae1.1, Pseudomonadaceae1.2; Genus: *Escherichia1.1, Klebsiella1.1*, P*seudômonas* 1.2.	
			PDAC			
					CCA: Phylum: Proteobacteria1; Family: Enterobacteriaceae1.1, Pseudomonadaceae1.2; Genus: *Escherichia1.1, Pseudômonas* 1.2	
Riquelme E (2019), USA	Cohort	16s rRNA	PDAC	Stool	LTS: Phylum: *Proteobacteria1, Actinobacteria2*; Class: *Xanthomonadaceae1, spretomycetales2.1, Pseudonocardiaceae2.2, Baccilaceae3*; Genus: Pseudoxanthomonas1, Streptomyces2.1, Saccharopolyspora2.2; bSpecies: *Bacillus clausii*	
					STS: Phylum: *Firmicutes1, Bacterioidetes2*; aClass: *Clostridia1, Bacteroide2*	

CCA, Cholangiocarcinoma; CBD stones, Common bile Duct Stones; GBC, Gallbladder Carcinoma; PDAC, Pancreatic Ductal Adenocarcinoma; LTS, Long Term Survival; STS, Short Term Survival.

a The study did not describe taxonomy at finer levels than the class, so a class was held for a comparison of the two groups.

b The study emphasized the importance of the presence or absence of this species in the result, even though the genus to which it belongs is not among the most prevalent.

**Table 5:** Objectives and conclusions of studies.

Author, publication date and country	Objectives	Conclusions
Chen B (2019), China	“To investigate whether the dCCA has a certain correlation with biliary microecology, and to detect specific strains”.	Microbiota of patients with dCCA differed significantly from those with lithiasis alone. Individuality in the biliary microbiota was found, per patient. Such information can be used to treat diseases of the biliary tract.
Vogtmann E (2020), USA	“We evaluated the association between oral microbiota and pancreatic cancer in Iran”.	The oral microbiota differed between cases and controls, with some bacterial rates being more abundant or present in the cases, and this may be related to the presence of cancer, or risk for development.
Half E (2019), Israel	“To examine the gut microbiome alterations in PC and their potential to serve as biomarkers”.	Given the difficulty of associating microbial patterns with cancer, this would become even more difficult in its early stages. An alternative would be to compare the microbial pattern with other non-invasive biomarkers.
Serra N (2018), Canada	“In this study, we aimed to assess the bile microbiological flora and its potential link with comorbidity in women”.	More analyses are needed to better understand the virulence of known pathogens and there may be an association between non-adherence to the Mediterranean diet and changes in the intestinal microbiota and bacteria.
Olson SH (2017), USA	“In this pilot study, we compared the oral microbiota in patients with newly diagnosed, untreated, PDAC, and healthy controls, hypothesizing that the oral microbiota would differ between cases and controls”.	There does not appear to be a strong relationship between the risk of PDAC and IPMN and oral microbiota, but differences in individual “rates” should be evaluated in larger studies, which also need to work on confounding variables of association between cases and controls.
Di Carlo P (2019), Italy	“To evaluate the effect of bile microbiota on survival in patients with Pancreas and Biliary Tract Disease (PBD)”.	Some bacteria isolated from bile samples can be considered risk factors for carcinogenesis and/or progression of diseases of the biliopancreatic tract, and this knowledge is important for the indication of antimicrobial therapies for these patients with PBD neoplasms.
Mei Q-X (2018), China	“In this study, our aim was to characterize the specific composition of the duodenal microbiota in pancreatic cancer patients using 16S ribosomal RNA (rRNA) pyrosequencing methods”.	The role of the duodenal microbiota in pancreatic head cancer cannot be ruled out, as the analysis of the microbiota based on LEfSe revealed small changes in relation to healthy control subjects.
Erick R (2019), USA	“To gain insights on the host-related influences that might guide this unusual long-term survival”.	The tumor microbiome has a powerful effect in determining PDAC survival. The unique LTS tumor microbiome may contribute to form a favorable tumor microenvironment, characterized by the recruitment and activation of CD8 T cells into the tumor milieu and may also be useful as a predictor of patient outcomes. The microbiome-based prognostic tool, results represent an opportunity to manipulate the microbiome to improve the life expectancy of patients with PDAC.
Torres PJ (2015), USA	“To determine the salivary profiles of patients with and without pancreatic cancer. The use of HTS to sequence 16S rRNA bacterial genes from entire salivary microbial communities allows for a more comprehensive profile of the microbiome in health and disease”.	There was a higher proportion of *Leptotrichia* in patients with pancreatic cancer, while the proportion of *Porphyromonas* and *Neisseria* was lower in these patients. More studies on the subject with a larger number of patients are needed to overcome biases.
Ren Z (2017), China	“Thus, it is hypothesized that gut microbiota is associated with PC but gut microbial characteristics in clinical PC have not been reported... The gut microbial composition, taxonomic difference, microbial function prediction and microbial markers were performed”.	Patients with CP showed reduced gut microbiota diversity and a unique microbial profile that differs from CH, partially attributed to decreased alpha diversity. Intestinal microbial changes in PC showed an increase in some potential pathogens and LPS-producing bacteria and a decrease in some probiotics and butyrate-producing bacteria. Changes in microbial gene functions were consistent with taxonomic changes in PC. Streptococcus has been associated with bile in the intestine.
Fan X (2018), USA	“Determine if oral microbiome was associated with subsequent risk of pancreatic cancer”.	The study demonstrates that transport of *P. gingivalis* and *A. actinomycetemcomitans* and decreased relative abundance of the phylum *Fusobacteria* and its genus *Leptotrichia* are related to an increased subsequent risk of pancreatic cancer. It also provides evidence that oral microbiota may play a role in the etiology of pancreatic cancer.
Kohi S (2021), EUA	“We tested the hypothesis that duodenal fluid may contain microbial alterations associated with PDAC”.	There are changes in the duodenal fluid microbiome and mycobiota in patients with PDAC that could be used to better stratify pancreatic cancer risk
Saab M (2021), France	“To investigate (...) a series of 30 extrahepatic CCA patients who underwent ERCP in an effort to identify the biliary microbiota signature”.	Given the significant differences between microbiota of patients with and without cancer, excluding comorbidities that could act as a possible confounding factor, CCA dysbiosis can help to identify patients with this cancer.
Sun H (2020), China	“We carried out this research to discover new available salivary biomarkers of PC, and to comprehensively explain the potential mechanism of oral microbes in the pathogenesis of PC”.	There is a difference between the oral microbiota of healthy people and those with pancreatic diseases (such as pancreatic cancer), but more study is needed to determine the causal relationship between the two situations.
Wei AL (2020), China	“To investigate the saliva microbiome distribution in patients with pancreatic adenocarcinoma (PDAC) and the role of oral microbiota profiles in detection and risk prediction of pancreatic cancer”.	The composition of the oral microbiome is different in PDAC and healthy individuals and this knowledge of the bacterial flora is important for developing treatments and reducing the risk of pancreatic cancer.

CCA, Cholangiocarcinoma; PC, Pancreatic Cancer; IPMN, Intraductal Papillary Mucinous Neoplasms; PBD, Pancreas and Biliary Tract Disease; PDAC, Pancreatic Ductal Adenocarcinoma; LTS, Long Term Survival; HTS, High-Throughput Sequencing; ERCP, Endoscopic Retrograde Cholangiopancreatography.

15 scientific papers were included, with a total of 2594 participants. The minimum number of participants in a study was 28 and the maximum was 732, 50% of the studies had at least 108 participants. The mean age of participants was 63.07 years (standard deviation 7.72). Most participants were male (53.8%). The results are shown in Table 6.

**Table 6:** Description of studies included in the systematic review.

Systematic review		
Total of studies		n =15
Total participants		n = 2594
Sample size (Total number of participants)	Mean	173
	Median (minimum‒maximum)	108 (28‒732)
Age of participants	Mean (standard deviation)	63.07 (7.72)
	Median (minimum‒maximum)	63.53 (44‒74)
Sex^a^	Male, n (%)	1290 (53.8%)
	Female, n (%)	1106 (46.2%)

a 25 participants from the Riquelme study lacked information.

The comorbidities listed in the articles mainly include diabetes mellitus, systemic arterial hypertension, and dyslipidemia. Riquelme E. et al.^26^ and Half, E. et al.^31^ include obstruction of the biliary tract (caused by the presence of tumor, calculi, thickening of walls, or unknown reasons) due to increased serum levels of canalicular enzymes. Other associated factors are alcohol consumption and smoking, increased serum creatinine and white blood cells, cholelithiasis, and increased ALT and AST, and direct and total bilirubin.

The selected articles were published between 2015 and 2021, with six studying oral microbiota; four biliary; three intestinal per fecal sample, and two per duodenal samples (tissue or fluid). Mostly, they compare a controlled microbiota and a patient previously diagnosed with pancreatic or biliary cancer. Selected reviews include countries: Canada, China, South Korea, United States, Finland, Israel, France, and Italy.

The selected studies mostly analyzed cases of PDAC (13 studies), followed by CCA,^4^ Gallbladder Carcinoma (GBC),^1^ each study being possible to include one or more types of neoplasm of the biliopancreatic tract. Participants in each of the studies were separated into groups for microbiome analysis. The case groups formed were according to the type of cancer of the patients (PDAC and CCA, for example) or, as in Riquelme E. et al.,^26^ survival time of the patient with the disease. Controls were divided into healthy or benign disease patients (calculi in bile ducts and Intraductal Papillary Mucinous Neoplasm ‒ IPMN, for example). The same study may have presented more than one case or control groups, such as Sun, H. et al.,^32^ who had one healthy control and one with benign conditions.

Of the 11 selected studies, 9 used 16s rRNA as an analysis method to characterize the specific composition of the microbiota. On the other hand, Serra, N. et al.^27^ and Di Carlo, P. et al.^28^ analyzed the biliary microbiota using the BD Phoenix System. In addition, Di Carlo used the Vitek-2 system together.

In studies in which the oral microbiota was analyzed, Vogtmann, E. et al.^29^ has as control microbiota a predominance of the genus *Haemophilus* (from the phylum *Proteobacteria*), whose presence would decrease the chance of biliopancreatic neoplasia (OR = 0.95), while, for the case group, there was an increase in the families *Lachnospiraceae* G7, *Bacteroidaceae, Staphylococcaceae* and *Enterobacteriaceae*, but does not specify the genus. Olson, S.H., et al.^33^ cites the presence of the genus *Neisseria* and *Haemophilus* (both from the phylum *Proteobacteria*) in the control group and the genus *Streptococcus* (phylum *Firmicutes*) in the case group. In both cases, the disease was PDAC.

The control cases of Fan, X. et al.,^22^ Wei, AL. et al.^34^ and Torres, P.J. et al.,^23^ diverge in relation to the results of the analyses of the oral microbiota. Fan, X. et al. associate the low risk of PDAC with the presence of bacteria of the genus *Leptotrichia* (phylum *Fusobacteria*) (95% CI 0.89 to 0.99; OR = 0.87 and 95% CI 0.79 to 0.95) and high risk with the presence of *Porphyromonas* (phylum *Bacteroidetes*) (OR for presence vs. absence = 1.60 and 95% CI 1.15 to 2.22; OR = 2.20) and *Aggregatibacter actinomycetemcomitans* (phylum *Proteobacteria*) (OR = 2.20 and 95% CI 1.16 to 4.18). Torres, P.J. et al. and Wei, AL. et al., however, reported a decrease in these same bacteria genera in patients with PDAC, *Porphyromonas* and *Neisseria*, and an increase in *Leptotrichia.* Wei, AL. et al. also found an increase in *Streptococcus*.

Sun, H. et al.,^32^ found the genus *Neisseria* among the most abundant in the oral microbiome mainly in controls, but also in cases of PDAC, in alignment with Wei, AL. et al. and Torres, P.J. et al. On the other hand, Wei, AL. et al. associates *Veillonella* with low risk for PDAC, Sun, H. et al. found this genus as one of the most prevalent in cases of the disease.

In the analysis of the biliary microbiome, two cross-sectional studies and one cohort reported an increase in *Escherichia* (phylum *Proteobacteria*, family *Enterobacteriaceae*) in patients with CCA. Serra, N. et al.,^27^ and Di Carlo, P. et al.^28^ found an increase in Pseudomonas (phylum *Proteobacteria*, family *Pseudomonadaceae*), while Chen, B. et al.,^25^ found *Klebsiella* (phylum *Proteobacteria*, family *Enterobacteriaceae*), in addition to *Faecalibacterium, Okibacterium* and *Corynebacterium*. The case and control groups (composed of patients with choledocholithiasis) by Chen, B. et al., showed an increase in *Escherichia*/*Shigella* and *Klebsiella*, and only the case group, *Halomonas, Streptococcus* and *Enterococcus*. The control case of Saab, M. et al. diverged from the other three with bile analysis, with the CCA group presenting the genera *Streptococcus, Pyramidobacter,* and *Bacteroidetes* as more abundant compared to the controls, with no congruence with the other studies.

Di Carlo, P. et al.,^28^ and Serra, N. et al.^27^ analyzed the biliary microbiome in patients with PDAC. The first reported *Escherichia* coli, *Klebsiella pneumoniae,* and *Pseudomonas aeruginosa* as more frequent in patients, in contrast to the second, which, despite having found *Klebsiella spp* as a positive predictor, showed *Escherichia spp* and *Pseudomonas spp* as negative predictors. In addition, Serra, N. et al. reported *Pseudomonas spp* as a positive predictor for GBC.

The four articles that analyze the intestinal microbiota point to *Firmicutes* as among the most frequently found phyla, in cases and controls. In two of them, *Proteobacteria* also appeared in both groups. Q.-X. Mei et al.,^30^ when comparing the duodenal microbiota of patients with PDAC with healthy controls, place the genera *Acinetobacter, Aquabacterium, Rahnella, Delftia, Sphingobium, Massilia, Oceanobacillus, Deinococcus* as more abundant in the first group than in the second, while *Escherichia, Shigella, Pseudomonas, Enhydrobacter, Porphyromonas, Paenibacillus* were less abundant. For Kohi, S., et al.,^35^ however, *Bifidobacterium, Enterococcus, Clostridium, Escherichia, Shiguella,* and *Fusobacterium* were the most abundant genera in PDAC when compared to a benign disease or healthy controls, even though it was also an analysis of the duodenal flora.

Riquelme E. et al.,^26^ a cohort, compared the intestinal microbiota between patients with short (STS) and long (LTS) survival after the diagnosis of PDAC, obtaining a result that in LTS patients there is a predominance of the phyla *Proteobacteria* (genus *Pseudoxanthomonas*) and *Actinobacteria* (genera *Streptomyces* and *Saccharopolyspora*), in addition to the presence of *Bacillus clausii*. In STS patients, however, there were no predominant genera, but classes: *Clostridia* and *Bacteroidea*, contrary to Kohi S., et al.,^35^ who found, for these, the *Fusobacterium, Rothia* and *Neisseria* genera as the most prevalent and belonging to classes that differ from those mentioned above by Riquelme E. et al. Half et al.^31^ and Ren et al.^24^ show agreement at the family level regarding the increase of *Veillonellaceae* in PDAC and at the genus level regarding the greater presence of *Clostridium* in controls compared to cases. Otherwise, there were no findings common to these studies.

## Discussion

To reduce the mortality of pancreatic and biliary tract cancer, it is important to have methods that help in the early diagnosis and intervention of the disease. For this, studies relating to microbiota, whether fecal, biliary, or oral, with the incidence of PDAC and CCA have great importance in the academic world. This systematic review analyzed 15 articles that assess the microbiota of patients with cancers of the biliopancreatic, comparing it or not with that of controls.

About 86.67% of the studies use 16s rRNA as a sequencing method to assess the composition of the microbiota. This method is useful when there is no basic knowledge about the possible findings of the analysis, in addition to being a well-known and lower-cost method compared to other techniques. Despite this, the method is not the most suitable for detecting strains for epidemiological purposes or as a specific virulence factor.^36^ The other method used to approach the microbial composition was BD Phoenix System, which is culture-dependent, assessing only pathological bacteria. Considering this, a proper comparison between studies with different analysis methods is not possible, for the intrinsic selection bias caused by the distinct perspective of which one of them. Another factor that should be taken into account regarding the use of 16s rRNA sequencing is the no specificity of the method to any particular group, without restricting the taxonomic classification to be used by the researcher. One of the greatest difficulties encountered in the systematic review was the heterogeneity of the presentation of results in relation to taxonomic groups. For example, Fan, X. et al.^22^ reports the most frequently found species, while Vogtmann, E. et al.^29^ cites a genus and some families present in greater abundance, making it difficult to compare them.

To reduce the differences between the studies and standardize them, in order to make comparison possible, only phylum, family, and gender were included in the tables present in the results, and class, when the previous ones had not been made available by the author. It was necessary to research and classify the proposed taxonomic phyla (phylum, family, and genus), which was not possible in those studies that selected broader categories. Considering the importance of comparing studies, these should place more than one classification in the microbiota found or it should be agreed that studies on microbiota and its possible pathogenicity always select the same classifications, for example, family or genus.

There was also great variety in the ways to expose the constitution of the microbiome obtained after the analyses. Some articles, such as Di Carlo, P. et al.,^28^ only indicate which strains are most prevalent in each group, while others, such as Olson, S. H. et al.,^33^ do not describe the samples individually, but only the differences between groups. This makes it difficult and sometimes impossible to understand the actual composition of each sample and establish a pattern considered healthy and another characteristic of each disease studied. Despite this, there are articles in which this exposition was complete, describing both the composition of the samples by group and between different groups, as in Q.-X. Mei et al.,^30^ is a good model for future articles addressing this topic.

Another point to be highlighted is the difficulty in comparing the results of case-control studies, which diverged in terms of the phyla and genera found. This can be attributed to the different nationalities of the studies, such as Half, E et. al.,^31^ who is Israeli, and of Ren Z. et. al.,^24^ who is Chinese. The geographic difference is also reflected in lifestyle habits and genomic factors. Furthermore, these studies also differ regarding the specification of comorbidities and the inclusion of patients with laboratory alterations, which were carried out only by Half, E. et al. In this study, healthy people with Non-Alcoholic Fatty Liver Disease (NAFLD) were included, with comorbidities such as diabetes mellitus (13%), systemic arterial hypertension (50%) and dyslipidemia (88%), factors that can influence the composition of the microbiome.^37^ Thus, it is not possible to determine the effects of this difference in the composition of the fecal microbiota.

Although there are main phyla present in the intestine (*Bacteroidetes* and *Firmicutes*),^38^ the collective microflora is composed of more than 35,000 bacterial species^39^ and it is difficult to determine a static control composition, since even primary pathogens that inhabit the human intestine, in low incidence and in symbiosis, are referred to as healthy.^40^ Thus, it was not possible to determine what is, and if there really is, a microbiota to be used as a control.

In the studies by Ren, Z et al.^24^ and Mei, Q-X et al.,^30^ the phylum *Proteobacteria* was found in abundance in cases and controls. Kohi, S. et al.^35^ also found this for controls but diverged from Hollister, B et al.,^40^ who found little abundance of this phylum in healthy individuals and an increase in cases of gastrointestinal tract disease. Furthermore, Hollister, B et al. proposed *Streptococcus* as the main genus in the non-diseased duodenum, while Mei Q-X et al. does not mention it and Kohi S et al. found an increase in this genus only in proton pump inhibitors users. These disagreements reinforce the need for additional studies to determine the composition of the human microbiota in its various sites, health conditions, and interfering factors.

With regard to the comparison of the oral microbiota, Fan, X. et al.,^22^ from New York, cited the presence of bacteria of the genus *Leptotrichia* as low risk for PDAC and *Porphyromonas* and *Aggregatibacter actinomycetemcomitans* as high risk. On the other hand, Torres, P.J. et al.,^23^ whose study was conducted in San Diego, concluded that the presence of *Porphyromonas* and *Neisseria* is linked to low risk of PDAC while *Leptotrichia* is linked to high risk. Both studies took place in the same country with similar populations (ethnicity, risk factors, age), which would tend to reduce the difference between results in the microbiota found. Despite this, their conclusions were contradictory, while Wei AL et al.,^34^ a Chinese study, showed agreement with Torres, P.J. et al., probably due to different sample collection methodologies, in which Fan, X. et al. collected saliva with mouthwash, while the other two collected the material without the use of other liquids. This reinforces the difficulty of comparing microbiota.

On the other hand, Vogtmann, E. et al.^39^ and Olson, S.H., et al.^33^ agree in citing the presence of the genus *Haemophilus* and its higher taxonomic levels as protective, despite approaching different populations, the first from the northeast of the USA and the second from Iran.

Bile was the analyzed sample that had the greatest agreement among the results. Serra, N. et al.^27^ and Di Carlo, P. et al.^28^ describe a positive correlation between the presence of *Klebsiella* and PDAC, but they diverge as to the role of the genera *Escherichia* and *Pseudomonas*, as the first classified them as negative predictors for the disease and the second as the most common genera. But this difference can be explained by the type of analysis carried out by Serra N. et al., who compared three different diseases (PDAC, CCA and GBC) and, based on this comparison, arrived at these results. But the similarity may be explained by the BD Phoenix System used for analysis, which restricts the searched composition to the clinically significant bacteria. This may also explain the difference between the latter and Chen. B et al.,^25^ for which the presence of *Klebsiella* is related to the appearance of CCA, but the investigation was made using 16S rRNA for sequencing.

The great disagreement regarding the microbiota among the selected articles can be explained by the various factors that influence it. Among them are age, hygiene, life habits, diet, and other external factors,^41^ therefore, it is possible to claim that there will always be a difference in the microbiome, especially in very different cultures. Establishing a consensus on the taxonomic description and obtaining samples is essential to allow comparison between results. For future studies that seek to assess the impact of cancer on the composition of the microbiome, the material should be collected at two different times: one before and one after the suspicion and diagnosis.

After analyzing the selected articles and given the limitations described, it is not possible to state that any microorganism can be related to pathogenicity, colonization, or used as screening for patients with PDAC or CCA.

In an attempt to more accurately characterize the biliary microbiota related to PDAC, there is work being carried out by the same team of this review that aims to analyze the difference between the microbiota of healthy people compared to patients with hepatobiliopancreatic diseases.^42^

## Conclusion

There was great disagreement in the characterization of both the microbiota of patients with benign diseases and patients with cancer of the biliopancreatic tract. The literature is still more focused on the study of the intestinal microbiota, with comparisons being made between healthy patients and those with PDAC. Thus, studies that analyze the microbiome of other sites, such as biliary and pancreatic, or its possible alterations in diseases such as CCA, are still scarce, making it difficult to adequately assess the data in this regard. In addition, the composition of the microbiota is greatly influenced by lifestyle habits and comorbidities, and it is questioned whether there really is a microbiota to be defined as normal. Due to these factors, it was not possible to find any specific marker or to associate any genus of microbiota bacteria with PDAC or CCA. More studies are needed not only to determine cancer-associated virulence factors but also to characterize healthy and pathogenic microbiota.

## Abbreviations

PDAC, Pancreatic Ductal Adenocarcinoma; CCA, Cholangiocarcinoma; NFKB, Nuclear Factor Kappa B; VEGF, Vascular Endothelial Growth Factor; PRISMA-P, Preferred Reports for Systematic Reviews and Protocol Meta-Analysis; PROSPERO, Prospective Register of Systematic Reviews; PICO, Patient or Problem, Interest, Control or Comparison, Outcome; IPMN, Intraductal Papillary Mucinous Neoplasms; PBD, Pancreas and Biliary tract Disease; LTS, Long Term Survival; STS, Short-Term Survival; HTS, High-Throughput Sequencing; ERCP, Endoscopic Retrograde Cholangiopancreatography; M, Male; F, Female; CBD, Common Bile Duct; RC, Recurrent Choledocholithiasis; PC, Pancreatic Cancer; PCL, Precancerous Lesions; NAFLD, Non-Alcoholic Fat Liver Disease; GBC, Gallbladder Carcinoma; DM, Diabetes Mellitus; AST, Aspartate Aminotransferase; ALT, Alanine Aminotransferase; Tbil, Total Bilirubin; Dbil, Direct Bilirubin; Scr, Serum Creatinine; WBC, White Blood Cell; NE, Neutrophilic Granulocyte.

## Ethics approval and consent to participate

Not applicable.

## Consent to publish

Not applicable.

## Availability of data and materials

All data produced and obtained is available within the manuscript.

## Authors' contributions

AM had full access to all the data in the study and take responsibility for the integrity of the data and the accuracy of the data analysis. Concept and design: VCM, FSN, MOS, AM. Acquisition, analysis, or interpretation of data: VCM, FSN, MOS, JVT, LZP, WAFM, VSC, WTH, LRI, MSK, LACDA, AM, WA. Drafting of the manuscript: LACD, VCM, FSN, MOS, WAFM. Critical revision of the manuscript for important intellectual content: AM, LRI. Statistical analysis: MOS, JVT, LZP, VSC, MBT. Administrative, technical, or material support: VCM, WAFM, WTH, LRI. Supervision: AM, LACDA, WA. All authors have read and approved the manuscript.

## Funding

No funding.

## Declaration of Competing Interest

The authors declare that they have no known competing financial interests or personal relationships that could have appeared to influence the work reported in this paper.

## References

[1] Instituto Nacional de Câncer José Alencar Gomes da Silva. Estimativa 2020: incidência de câncer no Brasil/Instituto Nacional de Câncer José Alencar Gomes da Silva. – Rio de Janeiro: INCA, 2019. Available at:https://www.inca.gov.br/sites/ufu.sti.inca.local/files/media/document/estimativa-2020-incidencia-de-cancer-no-brasil.pdfk. Access on 26th July 2020.

[2] Siegel RL, Miller KD, Jemal A. (2020). Cancer statistics, 2020. CA Cancer J Clin.

[3] Ferlay J, Ervik M, Lam F (2020). Global Cancer Observatory: Cancer Today.

[4] Ministério da Saúde. DATASUS. Available at: http://tabnet.datasus.gov.br/cgi/deftohtm.exe?sim/cnv/obt10uf.def. Access on 07th May 2020.

[5] Bridgewater JA, Goodman KA, Kalyan A, Mulcahy MF. (2016). Biliary tract cancer: epidemiology, radiotherapy, and molecular profiling. Am Soc Clin Oncol Educ Book.

[6] Torre LA, Siegel RL, Islami F, Bray F, Jemal A. (2018). Worldwide burden of and trends in mortality from gallbladder and other biliary tract cancers. Clin Gastroenterol Hepatol.

[7] Goggins M, Overbeek KA, Brand R, Syngal S, Del Chiaro M, Bartsch DK (2020). International Cancer of the Pancreas Screening (CAPS) consortium. Management of patients with increased risk for familial pancreatic cancer: updated recommendations from the International Cancer of the Pancreas Screening (CAPS) Consortium. Gut.

[8] Khan SA, Tavolari S, Brandi G. (2019). Cholangiocarcinoma: epidemiology and risk factors. Liver Int.

[9] Del Castillo E, Meier R, Chung M, Koestler DC, Chen T, Paster BJ (2019). The microbiomes of pancreatic and duodenum tissue overlap and are highly subject specific but differ between pancreatic cancer and noncancer subjects. Cancer Epidemiol Biomarkers Prev.

[10] Farrell JJ, Zhang L, Zhou H, Chia D, Elashoff D, Akin D (2012). Variations of oral microbiota are associated with pancreatic diseases including pancreatic cancer. Gut.

[11] Chen XZ, Wang R, Chen HN, Hu JK. (2015). Cytotoxin-associated gene a-negative strains of helicobacter pylori as a potential risk factor of pancreatic cancer: a meta-analysis based on nested case-control studies. Pancreas.

[12] Schulte A, Pandeya N, Fawcett J, Fritschi L, Risch HA, Webb PM (2015). Association between Helicobacter pylori and pancreatic cancer risk: a meta-analysis. Cancer Causes Control.

[13] Wei MY, Shi S, Liang C, Meng QC, Hua J, Zhang YY (2019). The microbiota and microbiome in pancreatic cancer: more influential than expected. Mol Cancer.

[14] Mima K, Nakagawa S, Sawayama H, Ishimoto T, Imai K, Iwatsuki M (2017). The microbiome and hepatobiliary-pancreatic cancers. Cancer Lett.

[15] Thomas RM, Jobin C. (2020). Microbiota in pancreatic health and disease: the next frontier in microbiome research. Nat Rev Gastroenterol Hepatol.

[16] Zhang Q, Ye M, Su W, Chen Y, Lou Y, Yang J (2020). Sphincter of Oddi laxity alters bile duct microbiota and contributes to the recurrence of choledocholithiasis. Ann Transl Med.

[17] Moher D, Shamseer L, Clarke M, Ghersi D, Liberati A, Petticrew M (2015). PRISMA-P Group. Preferred reporting items for systematic review and meta-analysis protocols (PRISMA-P) 2015 statement. Syst Rev..

[18] University of Illinois at Chicago's Library of the Health Sciences at Peoria [Internet]. Evidence based medicine ‒ what is the PICO model? [cited 2020Sep23]. Available from: https://researchguides.uic.edu/c.php?g=252338&p=3954402.

[19] Ouzzani M, Hammady H, Fedorowicz Z, Elmagarmid A. (2016). Rayyan-a web and mobile app for systematic reviews. Syst Rev.

[20] National Heart, Lung, and Blood Institute. Study Quality Assessment Tools. Available at https://www.nhlbi.nih.gov/health-topics/study-quality-assessment-tools. Access on 26th July 2020.

[21] Cochrane Methods Screening and Diagnostic Tests [Internet]. Handbook for DTA Reviews. [cited 2020Sep23]. Available from: http://methods.cochrane.org/sdt/handbook-dta-reviews.

[22] Fan X, Alekseyenko AV, Wu J, Peters BA, Jacobs EJ, Gapstur SM (2018). Human oral microbiome and prospective risk for pancreatic cancer: a population-based nested case-control study. Gut.

[23] Torres PJ, Fletcher EM, Gibbons SM, Bouvet M, Doran KS, Kelley ST (2015). Characterization of the salivary microbiome in patients with pancreatic cancer. PeerJ.

[24] Ren Z, Jiang J, Xie H, Li A, Lu H, Xu S (2017). Gut microbial profile analysis by MiSeq sequencing of pancreatic carcinoma patients in China. Oncotarget.

[25] Chen B, Fu SW, Lu L, Zhao H. (2019). A Preliminary study of biliary microbiota in patients with bile duct stones or distal cholangiocarcinoma. Biomed Res Int.

[26] Riquelme E, Zhang Y, Zhang L, Montiel M, Zoltan M, Dong W (2019). Tumor microbiome diversity and composition influence pancreatic cancer outcomes. Cell.

[27] Serra N, Di Carlo P, Gulotta G, d' Arpa F, Giammanco A, Colomba C (2018). Bactibilia in women affected with diseases of the biliary tract and pancreas. A STROBE guidelines-adherent cross-sectional study in Southern Italy. J Med Microbiol.

[28] Di Carlo P, Serra N, D'Arpa F, Agrusa A, Gulotta G, Fasciana T (2019). The microbiota of the bilio-pancreatic system: a cohort, STROBE-compliant study. Infect Drug Resist.

[29] Vogtmann E, Han Y, Caporaso JG, Bokulich N, Mohamadkhani A, Moayyedkazemi A (2020). Oral microbial community composition is associated with pancreatic cancer: a case-control study in Iran. Cancer Med.

[30] Mei QX, Huang CL, Luo SZ, Zhang XM, Zeng Y, Lu YY. (2018). Characterization of the duodenal bacterial microbiota in patients with pancreatic head cancer vs. healthy controls. Pancreatology.

[31] Half E, Keren N, Reshef L, Dorfman T, Lachter I, Kluger Y (2019). Fecal microbiome signatures of pancreatic cancer patients. Sci Rep.

[32] Sun H, Zhao X, Zhou Y, Wang J, Ma R, Ren X (2020). Characterization of oral microbiome and exploration of potential biomarkers in patients with pancreatic cancer. Biomed Res Int.

[33] Olson SH, Satagopan J, Xu Y, Ling L, Leong S, Orlow I (2017). The oral microbiota in patients with pancreatic cancer, patients with IPMNs, and controls: a pilot study. Cancer Causes Control.

[34] Wei AL, Li M, Li GQ, Wang X, Hu WM, Li ZL (2020). Oral microbiome and pancreatic cancer. World J Gastroenterol.

[35] Kohi S, Macgregor-Das A, Dbouk M, Yoshida T, Chuidian M, Abe T (2020). Alterations in the duodenal fluid microbiome of patients with pancreatic cancer. Clin Gastroenterol Hepatol.

[36] Clarridge JE (2004). Impact of 16S rRNA gene sequence analysis for identification of bacteria on clinical microbiology and infectious diseases. Clin Microbiol Rev.

[37] Mazidi M, Rezaie P, Kengne AP, Mobarhan MG, Ferns GA. (2016). Gut microbiome and metabolic syndrome. Diabetes Metab Syndr.

[38] Jandhyala SM, Talukdar R, Subramanyam C, Vuyyuru H, Sasikala M (2015). Nageshwar Reddy D. Role of the normal gut microbiota. World J Gastroenterol.

[39] Frank DN, St Amand AL, Feldman RA, Boedeker EC, Harpaz N, Pace NR. (2007). Molecular-phylogenetic characterization of microbial community imbalances in human inflammatory bowel diseases. Proc Natl Acad Sci U S A.

[40] Hollister EB, Gao C, Versalovic J. (2014). Compositional and functional features of the gastrointestinal microbiome and their effects on human health. Gastroenterology.

[41] Ewaschuk JB, Dieleman LA. (2006). Probiotics and prebiotics in chronic inflammatory bowel diseases. World J Gastroenterol.

[42] Nascimento FSD, Suzuki MO, Taba JV, de Mattos VC, Pipek LZ, D'Albuquerque EMC (2020). Analysis of biliary MICRObiota in hepatoBILIOpancreatic diseases compared to healthy people [MICROBILIO]: Study protocol. PLoS One.

